# Hypoxia‐inducible factor‐1α attenuates renal podocyte injury in male rats in a simulated high‐altitude environment by upregulating Krüppel‐like factor 4 expression

**DOI:** 10.1113/EP091443

**Published:** 2024-05-22

**Authors:** Zeng Xiaoshan, Cheng Huan, Gan Zhilin, Mo Liwen, Zeng Yan, Cheng Yue

**Affiliations:** ^1^ College of Medicine Southwest Jiaotong University Chengdu PR China; ^2^ Department of Nephrology General Hospital of Western Theater Command of PLA Chengdu PR China

**Keywords:** high‐altitude, hypoxia‐inducible factor‐1α, Krüppel‐like factor 4, podocyte

## Abstract

Previous studies have shown that podocyte injury is involved in the development of proteinuria in rats under hypobaric hypoxia conditions. Prolyl hydroxylase inhibitors (PHIs) may reduce proteinuria. This study aimed to further investigate whether the protective effects of hypoxia‐inducible factor 1α (HIF1α) on podocyte injury induced by hypobaric hypoxia are related to Krüppel‐like factor 4 (KLF4). Rats were housed in a low‐pressure oxygen chamber to simulate a high‐altitude environment (5000 m), and a PHI was intraperitoneally injected. Urinary protein electrophoresis was performed and the morphology of the podocytes was observed by electron microscopy. Rat podocytes were cultured under 1% O_2_, and siRNA was used to interfere with KLF4 expression. The protein expression levels of HIF1α, KLF4, CD2‐associated protein (CD2AP) and nephrin were determined by western blotting. Compared with those in the experimental group, the rats in the intervention group on day 14 had lower urinary protein levels, increased protein expression levels of CD2AP and nephrin, and reduced podocyte injury. The results of in vitro experiments showed that the protein expression levels of KLF4, CD2AP and nephrin were greater in the PHI intervention group and lower in the HIF1α inhibitors group than in the low‐oxygen group. The protein expression of CD2AP and nephrin in the siKLF4‐transfected podocytes treated with PHI and HIF1α inhibitors did not differ significantly from that in the low‐oxygen group. HIF1α may be involved in reducing progressive high‐altitude proteinuria by regulating KLF4 expression and contributing to the repair of podocyte injury induced by hypobaric hypoxia.

## Abstract (highlights)

- **What is the central question of this study?**
  Podocyte injury is involved in the development of proteinuria in rats under simulated high‐altitude environments and prolyl‐hydroxylase inhibitors (PHI) may have a potential role in reducing proteinuria: what are the effects of PHIs on podocytes and what are the mechanisms?
- **What is the main finding and its importance?**
  PHIs may have a potential role in reducing proteinuria by upregulating local hypoxia‐inducible factor 1α (HIF1α) expression in the kidney to alleviate podocyte injury. HIF1α plays a potential role in regulating Krüppel‐like factor 4 expression and contributing to the repair of podocyte injury induced by hypobaric hypoxia.

## 1 INTRODUCTION

Some individuals who rapidly ascend to high altitudes develop proteinuria within a day or up to a month later (Rennie et al., 1970; Hansen et al., 1994; Lewis et al., 2015), and some can even develop acute kidney injury (Gilbert‐Kawai et al., 2016). Our previous studies have shown that proteinuria occurs in rats in a simulated high‐altitude environment of 5000 m. Along with proteinuria, podocyte lesions such as swelling and foot process fusion were detected, indicating that the hypobaric and hypoxic environment at high altitudes can cause some degree of podocyte injury in rats, which may contribute to the development of proteinuria in high‐altitude regions. However, prolonging the simulated high‐altitude feeding time resulted in an initial increase and then a decreasing trend in urinary protein levels, indicating an adaptive regulatory mechanism in response to acute hypobaric hypoxia‐induced proteinuria (Zeng et al., 2022).

Hypoxia‐inducible factor 1α (HIF1α) is an important transcription factor involved in hypoxic adaptation (Rankin & Giaccia, 2016) and is closely related to high‐altitude adaptation (Liu et al., 2007). Evidence suggests that HIF1α can increase renal tolerance to hypoxia or ischaemia and reduce renal ischaemia‒reperfusion injury (Kapitsinou et al., 2012; Ma et al., 2009; Schödel et al., 2009). Our previous study also indicated that rats exposed to a low‐pressure oxygen chamber showed a significant decrease in proteinuria and reduced podocyte damage after intervention with a proline hydroxylase inhibitor (PHI). However, the exact mechanism is not known (Zeng et al., 2022).

The Krüppel‐like factor (KLF) family of transcription factors, which have zinc finger structures, participate in the regulation of various cellular physiological processes (Chang et al., 2017; Rane et al., 2019). Among them, KLF4 is expressed in podocytes of the kidney and is closely related to the occurrence of proteinuria (Hayashi et al., 2014). KLF4 is renoprotective in the setting of acute kidney injury and is a critical regulator of proteinuria in mice and humans. KLF4 was shown to act on the TGF‐β signalling pathway and contribute to the high‐altitude adaptation of Tibetan pigs (Wang et al., 2020). Researchers have shown that hypoxia induces high expression of KLF4 in different cells, such as human aortic smooth muscle cells (Shan et al., 2020) and pulmonary arteriole smooth muscle cells, in mice with hypoxia‐induced pulmonary hypertension and pulmonary hypertension patients (Sheikh et al., 2015). Further analysis revealed that HIF1α‐induced platelet‐derived growth factor (PDGF)‐BB derived from endothelial cells contributes to enhanced KLF4 levels in smooth muscle cells.

Therefore, we postulate that the increase in HIF1α expression induced by hypobaric hypoxia at high altitudes may affect the expression of KLF4, which participates in the repair of podocyte lesions and reducing proteinuria.

## 2 METHODS

### 2.1 Ethical approval

This study was approved by the Animal Welfare Committee of General Hospital of Western Theater Command of PLA (ethics number: 2021EC3‐47), in accordance with the *Regulation on the Administration of Laboratory Animals*, approved by the State Council of the People's Republic of China (CLI.2.293192), which conforms with *Guide for the Care and Use of Laboratory Animals* of the US National Institutes of Health (8th edition, revised 2011). Furthermore, the experiments conformed to the principles and standards of reporting in animal experiments of *Experimental Physiology* (Grundy, 2015). All efforts were made to minimize the pain and suffering of the animals.

### 2.2 Animals

Seventy‐two specific pathogen‐free (SPF)‐grade male Sprague‒Dawley (SD) rats (Enwell Experimental Animal Co., Ltd, Chengdu, China, Certificate No. SCXK (Xiang) 2019‐0004) were used in this study. The rats were randomly allocated to three groups: the control group (*n* = 24), experimental group (*n* = 24) and intervention group (*n* = 24). Each group of rats was further randomized into four subgroups according to the time of sacrifice: 0, 7, 14 and 28 days, with six rats in each subgroup.

The rats in the experimental group and intervention group were housed in a low‐pressure oxygen chamber to simulate a high‐altitude environment (5000 m). The rats in the experimental group and intervention group were maintained under a 12‐h light–dark cycle with free access to water and food, an atmospheric pressure of 52.7–54.7 kPa, an oxygen partial pressure of 11.3–11.4 kPa, a temperature of 22–26°C, and a relative humidity of 50%–70%. The rats in the control group were housed in a normal pressure and normal oxygen environment (simulated altitude of 500 m, i.e., the average altitude of Chengdu, China) with a room temperature of 22–26°C and a relative humidity of 50–70%. The rats were also maintained under a 12‐h light–dark cycle with free access to water and food. PHI was intraperitoneally injected. The dose of l‐mimosine (Sigma‐Aldrich, St Louis, MO, USA) in the intervention group was 50 mg/kg, and l‐mimosine was intraperitoneally injected every other day. The rats in the control group and experimental group were given the same volume of physiological saline by intraperitoneal injection every other day.

The route of anaesthesia was intraperitoneal injection of 1% sodium pentobarbital (30 mg/kg). The animals were euthanized by decapitation approximately 30 min after anaesthesia induction.

### 2.3 Cell culture

Rat podocytes were purchased from BeNa Culture Collection(Suzhou, China) and cultured in Dulbecco's modified Eagle's medium (DMEM) supplemented with 10% fetal bovine serum (FBS) and 1% antibiotics (10,000 units/mL penicillin and 10,000 μg/mL streptomycin) at 37°C and 5% CO_2_. According to the experimental design, podocytes were divided into four groups: normoxia, hypoxia, hypoxia + PHI (l‐mimosine, Sigma‐Aldrich, 200 μmol/L), and hypoxia + HIF1α inhibitor (kc7f2, Selleck, Houston, Texas, USA, 40 μmol/L). The normoxia group was cultured in a normal air atmosphere with 21% O_2_ and 5% CO_2_, remainder N_2_. The hypoxia and hypoxia drug‐treated groups were cultured in a hypoxic atmosphere with 1% O_2_ and 5% CO_2_, and the remaining atmosphere was N_2_. The expression of the *KLF4* gene in podocytes was inhibited using siRNA interference technology, and after 48 h of culture, podocytes from each group were collected for western blot analysis to measure the protein expression levels of HIF1α, KLF4, CD2‐associated protein (CD2AP) and nephrin.

### 2.4 Collection of urine and kidney tissue specimens

Rats were placed in metabolic cages on days 0, 7, 14 and 28, and 24‐h urine samples were collected. The supernatant was extracted by centrifugation, and the protein concentration was determined by the BCA method. The distribution of proteins with different molecular masses in the urine was analysed by SDS‐PAGE, and all bands between 63 and 75 kDa were quantified. After urine collection, anaesthesia was induced using 1% sodium pentobarbital (30 mg/kg). Once anaesthesia was achieved, both kidneys were removed, and the animal was immediately euthanized by decapitation. Kidney tissue was collected as follows: a portion of kidney tissue was removed and fixed with 4% paraformaldehyde for immunohistochemical staining; a part of the renal cortex was fixed in 2.5% glutaraldehyde buffer and stored at 4°C for electron microscopy examination; and the remaining kidney tissue was placed in a cryopreservation tube, frozen in liquid nitrogen, and then frozen at −80°C for Western blot analysis.

### 2.5 Western blot

Western blotting was employed for the analysis of proteins. Total protein was extracted from kidney tissue and cultured cells using a whole protein extraction kit, and the protein concentration was determined using a BCA assay. Protein samples were separated by 8% SDS‐PAGE, followed by electroblotting onto polyvinylidene difluoride (PVDF) membranes. After transfer, the PVDF membranes were blocked with 5% fat‐free milk, and the following primary antibodies were added and incubated overnight at 4°C: rabbit anti‐HIF1α (Abcam, Waltham, MA, USA, no. ab179483, concentration 1:1000), rabbit anti‐KLF4 (Abcam, no. ab214666, concentration 1:1000), rabbit anti‐CD2AP (Bioss, Beijing, China, no. bs‐0512R, concentration 1:1000), and rabbit anti‐nephrin (Bioss, bs‐4866R, concentration 1:1000). β‐Actin (Proteintech, Rosemont, IL, USA, 20536‐1‐AP, 1:3000) was used as a loading control for the total proteins. The next day, secondary antibody was added and incubated for 1 h at room temperature (a goat anti‐rabbit antibody; Beyotime, Shanghai, China, no. 102419191119, concentration 1:3000). After washing, the membranes were incubated with a detection reagent (Millipore (Burlington, MA, USA) detection kit) at a dose of 400 U per membrane, the detected bands were visualized using the Bio‐Spectrum4 gel imaging system (Bio‐Rad, California, USA), and the band grey values were quantified by ImageJ software.

### 2.6 Immunohistochemistry

Renal tissues were fixed in 4% paraformaldehyde overnight, dehydrated in an ethanol gradient, cleared with xylene, embedded in paraffin and sectioned (thickness, approximately 4 μm). The tissue specimens were deparaffinized at room temperature, followed by antigen retrieval with EDTA. The membranes were blocked and incubated overnight at 4°C with the following primary antibodies (1:500 dilution): rabbit anti‐CD2AP (Bioss, Beijing, China) and rabbit anti‐nephrin (Bioss, Beijing, China). The next day, the specimens were incubated with secondary horseradish peroxidase‐conjugated goat anti‐rabbit (IgG H+L) antibodies (1:50 dilution) (Beyotime) in the dark. 3,3′‐Diaminobenzidine staining was then performed, followed by counterstaining with haematoxylin, dehydration and mounting of the slides. The specimens were observed and photographed under a microscope.

### 2.7 Transmission electron microscopy

The kidney tissue was initially fixed with 3% glutaraldehyde and further fixed with 1% osmium tetroxide. The specimens were gradually dehydrated with acetone and embedded in Ep812, and semithin sections were stained with toluidine blue for light microscopy localization. Ultrathin sections were then prepared using a diamond knife and stained with uranyl acetate and lead citrate. Transmission electron microscopy imaging was performed using a JEM‐1400FLASH microscope (JEOL, Japan).

### 2.8 siRNA transfection into cells

Cells in the logarithmic growth phase were digested, prepared as a cell suspension, and counted. The cell density was adjusted to 1 × 10^5^ cells/mL, and the cells were seeded into a six‐well plate. For the siRNA dilution solution, 250 μL of serum‐free medium and 100 pmol of siRNA were added and gently mixed to dilute the siRNA. In another centrifuge tube, 250 μL of serum‐free medium and 5 μL of Lip2000 were added and gently mixed to form a Lip2000 dilution. The diluted siRNA and diluted Lip2000 were mixed gently to form an siRNA–Lip2000 complex. The siRNA–Lip2000 complex was added to the seeded cells, and the plate was shaken to disperse the complex evenly. After 4 h of incubation in a 37°C CO_2_ incubator, the culture medium was replaced, and the cells were incubated for another 36 h. The cells were collected for subsequent experiments. Negative control siRNA (siRNA‐NC) (UUC UCC GAA CGU GUC ACG UdTdT), small interfering (siRNA)–KLF4‐1 (GGU CAU CAG UGU UAG CAA AdTdT), small interfering (siRNA)–KLF4‐2 (GGU GCA GCU UGC AGC AGU AdTdT), small interfering (siRNA)–KLF4‐3 (CGA AGA GUU CUC AUC UCA AdTdT), the plasmid vector and plasmid–KLF4 were purchased from Shanghai HanBio Co., Ltd (Shanghai, China).

### 2.9 Statistical analysis

Statistical analysis was conducted using SPSS 18.0 software (SPSS Inc., Chicago, IL, USA). Descriptive statistics for continuous variables are expressed as the means ± standard deviations after normality and homogeneity of variance were assessed. If the continuous variables followed a normal distribution with homogenous variances, one‐way analysis of variance (ANOVA) was applied to compare means among multiple groups, followed by pairwise comparisons between groups using a Bonferroni correction. Conversely, if the continuous variables did not follow a normal distribution or had heterogeneous variances, the Kruskal‒Wallis H‐test was used to identify differences in medians among multiple groups, followed by pairwise comparisons between groups using the Mann‒Whitney *U*‐test and a Bonferroni correction for *P*‐values. A *P*‐value less than 0.05 was considered to indicate statistical significance.

## 3 RESULTS

### 3.1 PHI alleviates proteinuria induced by low pressure and low oxygen in rats

**FIGURE 1:**
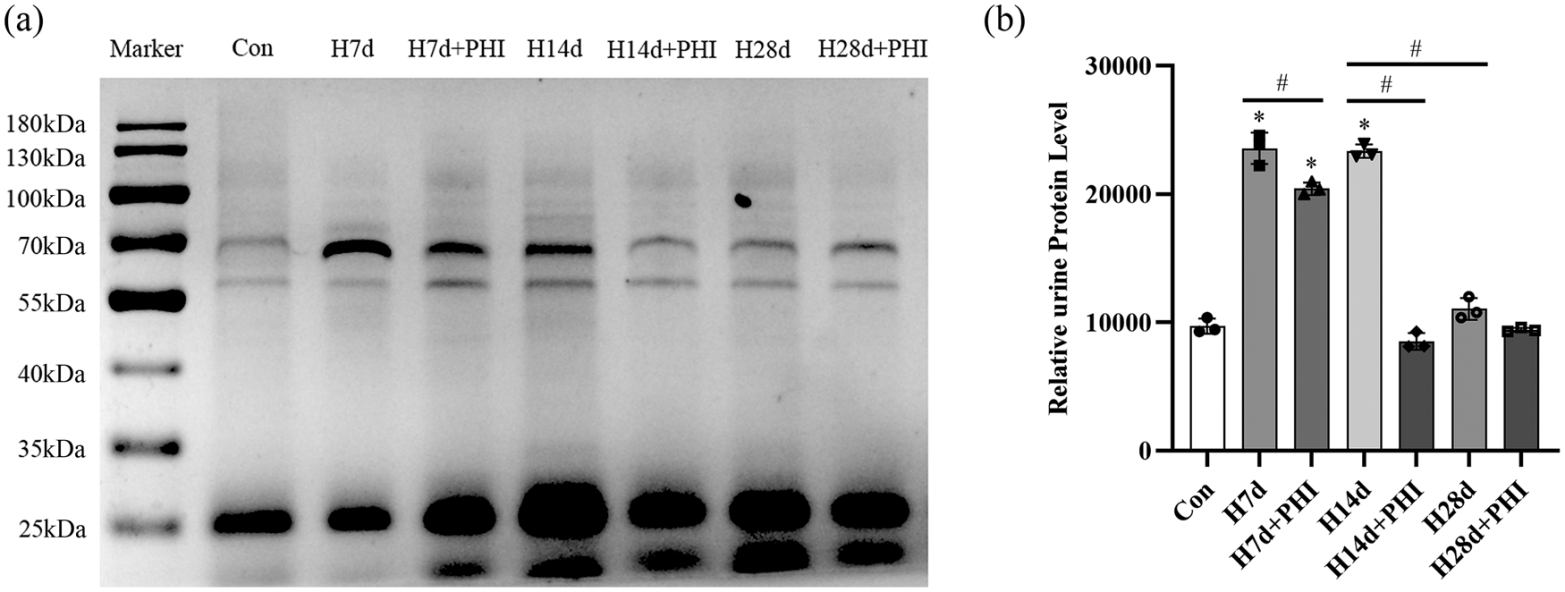
Urinary protein electrophoresis results for each group of rats. (a) Electrophoresis analysis results of rat urine protein. Con, control group; H7d, experimental group on day 7; H7d+PHI, intervention group on day 7; H14d, experimental group on day 14; H14d+PHI, intervention group on day 14; H28d, experimental group on day 28; H28d+PHI, intervention group on day 28. (b) The relative expression levels of urinary proteins with a molecular mass of 55‐70 kDa. The asterisk (*) indicates comparison with control group. *n* = 3. Data are expressed as means ± SD.

SDS‐PAGE analysis showed that in addition to small molecular mass proteins (with a molecular mass less than 25 kDa), medium molecular mass proteins (with a molecular mass of approximately 70 kDa) appeared in the urine of the rats in the experimental group. The urinary levels of medium molecular mass proteins in the experimental group significantly increased on days 7 and 14 compared to those in the control group (*P *< 0.0001). The urinary levels of medium molecular mass proteins on day 28 were significantly lower than those on day 14 in the experimental group (*P *< 0.0001). After PHI intervention, the levels of medium molecular mass proteins in the urine samples of the intervention group on days 7 (*P *= 0.0012) and 14 (*P *< 0.0001) were significantly lower than those in the urine samples of the experimental group at the same time points (Figure 1).

### 3.2 PHI alleviates foot cell damage caused by hypoxia and low pressure

**FIGURE 2:**
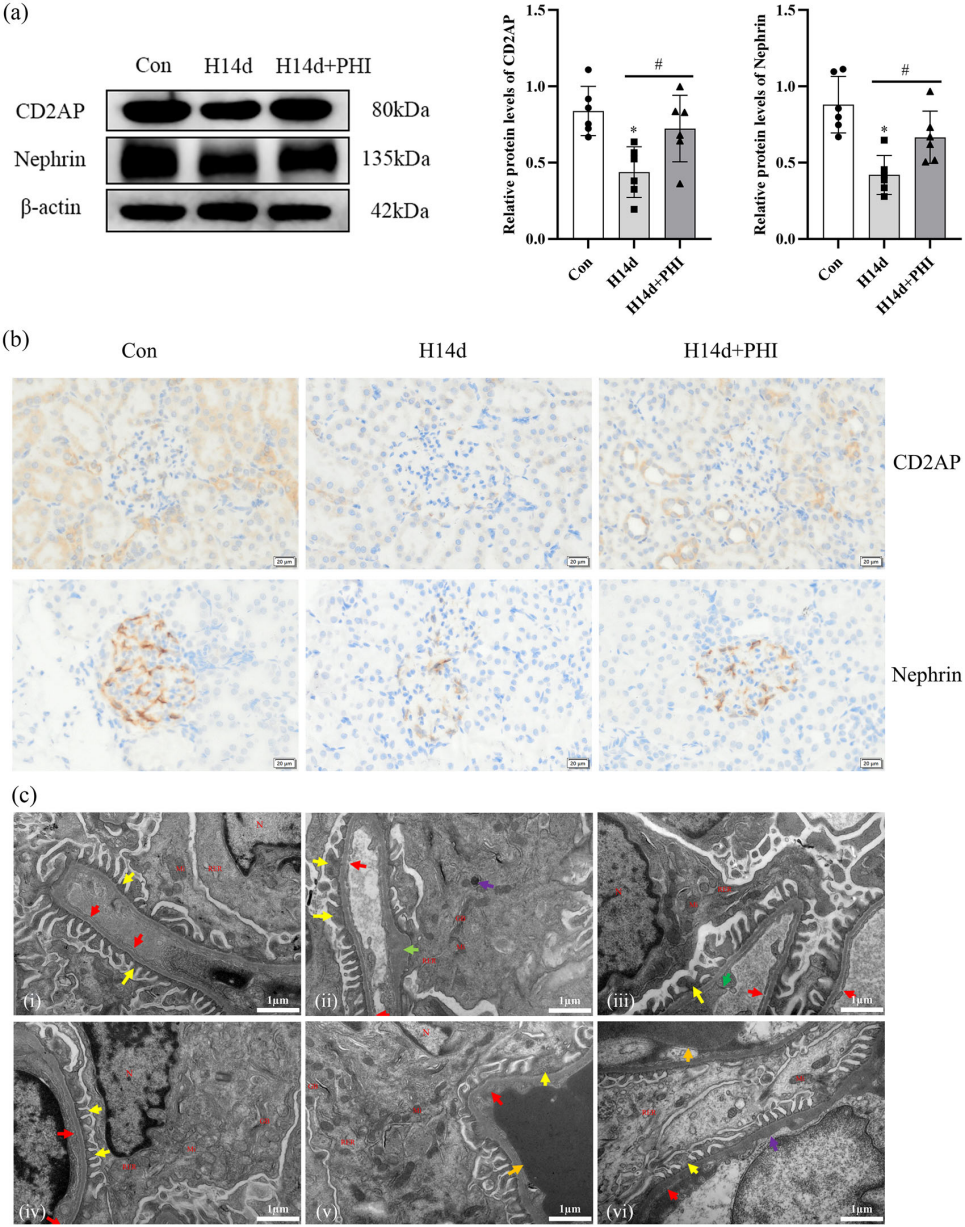
(a) Relative expression of rat podocyte marker proteins CD2AP and nephrin detected by western blot. The asterisk (*) indicates comparison with control group. The # symbol indicates comparison with different groups. *n* = 6. Data are expressed as means ± SD. (b) Relative expression of rat podocyte marker proteins CD2AP and nephrin detected by immunohistochemistry. Con, control group; H14d, experimental group on day 14; H14d+PHI, intervention group on day 14. (c) Transmission electron microscopy analysis rat glomerular and podocyte structures. (i, iv) control. (ii) Experimental group on day 14: autophagy (purple arrow), podocyte fusion (yellow arrow), basement membrane thickening (green arrow). (iii) Intervention group on day 14: podocyte fusion (yellow arrow). (v) Experimental group on day 28: podocyte fusion (yellow arrow), vascular lumen occlusion (orange arrow), endothelial window pore fusion (red arrow). (vi) Intervention group on day 28: peduncle fusion (yellow arrow), basement membrane thickening (purple arrow), vascular lumen occlusion (orange arrow), endothelial window pore fusion (red arrow). Nucleus (N), mitochondria (Mi), rough endoplasmic reticulum (RER), Golgi apparatus (GB). ×25,000.

Kidney tissue specimens were collected for western blot analysis and histopathological examination on the 14th day because the increase in the levels of medium molecular mass proteins in the urine on day 14 was most significant. The western blot results showed that the protein expression levels of CD2AP (*P *= 0.0048) and nephrin (*P *= 0.0005) in the renal tissues of the rats in the experimental group were lower than those in the renal tissues of the rats in the control group. The protein expression levels of CD2AP (*P *= 0.0416) and nephrin (*P *= 0.0471) in the renal tissues of the intervention group were greater than those in the renal tissues of the experimental group (Figure 2a). Immunohistochemical analysis was used to determine the protein expression of CD2AP and nephrin in renal tissue. CD2AP was expressed in both the glomeruli and tubules of the kidney, while nephrin was only expressed in the glomeruli. The control group showed high expression of CD2AP and nephrin, whereas the experimental group had significantly lower expression of both proteins than the control group, and the expression of CD2AP and nephrin in the intervention group was greater than that in the experimental group (Figure 2b). Electron microscopic observation of the glomeruli and podocytes revealed that the podocytes and foot processes in the control group were clearly intact with a continuous basement membrane of uniform thickness. However, in the experimental group, fusion of foot processes was observed with some regions of increased basement membrane thickness, and cytoplasmic autophagy was visible in podocytes. In the intervention group, the majority of organelles in the podocytes were normal, a few areas exhibited foot process fusion, and the basement membrane was complete and continuous, with good vascular patency and an intact endothelial cell structure (Figure 2c).

### 3.3 Hypobaric hypoxia increases the protein expression of HIF1α and KLF4 in rat kidney tissues

**FIGURE 3:**
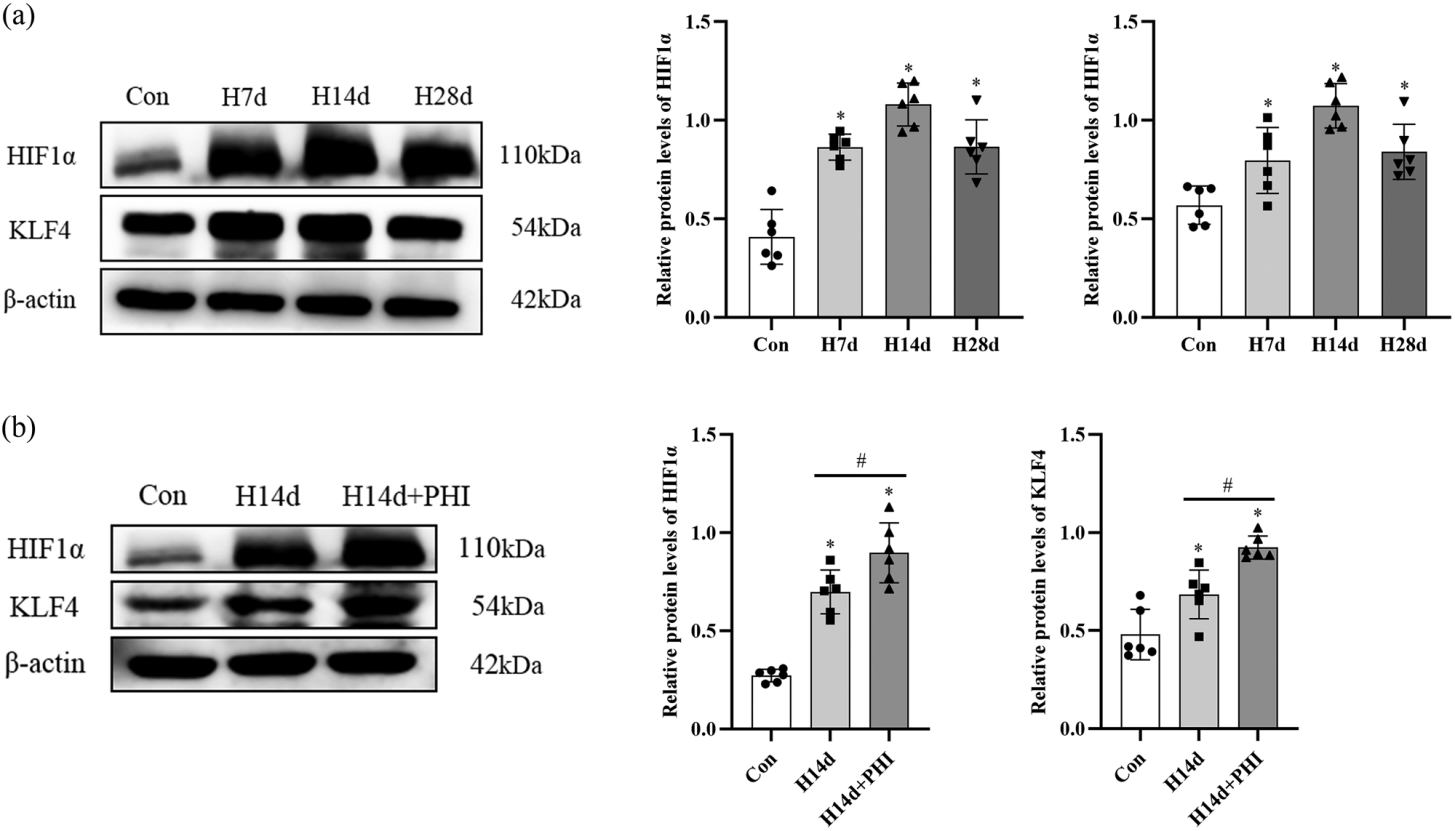
Relative expression of rat podocyte marker proteins HIF1α and KLF4 detected by western blot. Con, control group; H7d, experimental group on day 7; H14d, experimental group on day 14; H28d, experimental group on day 28; H14d+PHI, intervention group on day 14. The asterisk (*) indicates comparison with control group. The # symbol indicates comparison with different groups. *n* = 6. Data are expressed as means ± SD.

The western blot results showed that the protein expression of HIF1α in the kidney tissues of the rats in the control group was relatively low. On the seventh day of low‐pressure oxygen chamber feeding, the rats in the experimental group exhibited greater HIF1α protein expression in the renal tissues than did those in the control group (*P *< 0.0001). This high level of HIF1α protein persisted on the 14th day (*P *< 0.0001) and decreased on the 28th day but remained higher than that in the control group (*P *< 0.0001). KLF4 protein expression was detectable in the renal tissues of the rats in the control group under normal oxygen conditions. However, on the seventh (*P *= 0.0347), 14th (*P *< 0.0001) and 28th (*P *= 0.01) days of low‐pressure oxygen chamber feeding, the KLF4 protein expression in the renal tissues of the rats in the experimental group was significantly greater than that of the rats in the control group, and the most significant increase in KLF4 protein expression was observed on the 14th day (Figure 3a). With PHI intervention, the protein expression of HIF1α in the renal tissues of the rats in the intervention group increased significantly compared to that in the renal tissues of the experimental group (*P *= 0.0179), and the protein expression of KLF4 also increased significantly in the intervention group compared to that in the experimental group (*P *= 0.0047) (Figure 3b).

### 3.4 Podocyte injury and the protein expression of HIF1α and KLF4 under hypoxic culture conditions

**FIGURE 4:**
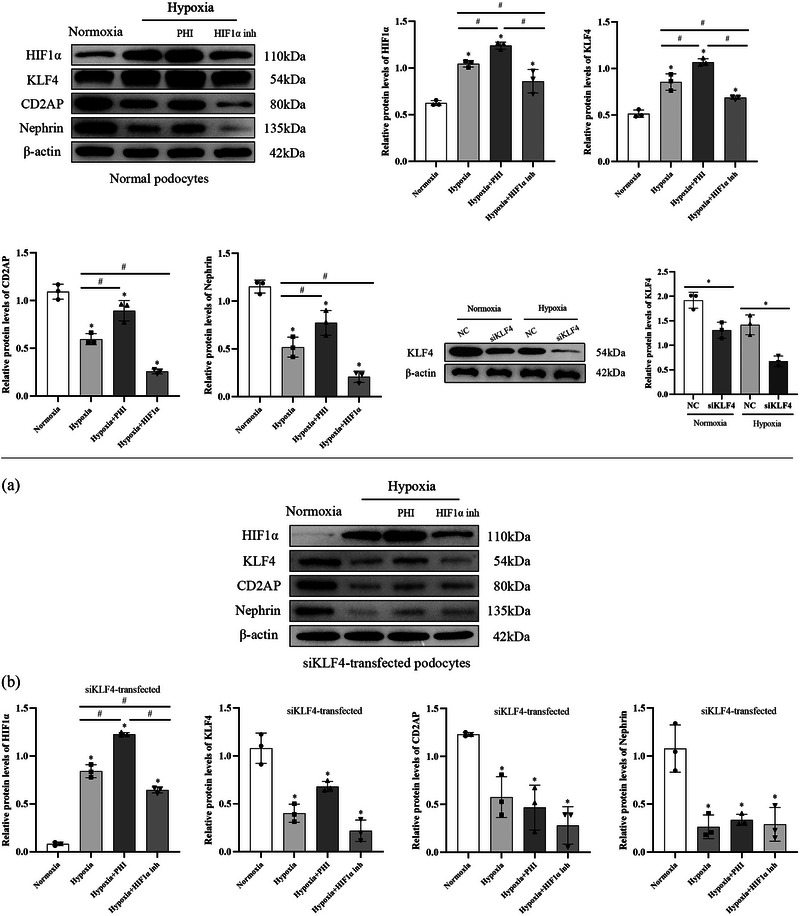
Relative expression of HIF1α, KLF4, CD2AP and nephrin proteins in normal podocytes of each group: Normoxia group, Hypoxia group, Hypoxia + PHI group; Hypoxia + HIF1α inhibitor group. The asterisk (*) indicates comparison with normoxia group. The # symbol indicates comparison with different groups. *n* = 3. Data are expressed as means ± SD.

Compared to those in the normoxic group, the protein expression levels of HIF1α (*P *= 0.0003) and KLF4 (*P *= 0.0002) were significantly increased in the podocytes of the hypoxic group, while the protein expression levels of CD2AP (*P *= 0.0002) and nephrin (*P *= 0.0002) were decreased. Compared to those in the hypoxic group, podocytes cultured under low oxygen conditions supplemented with PHI for 48 h exhibited a more significant increase in HIF1α protein expression (*P *= 0.0372) and KLF4 protein expression (*P *= 0.0048). Furthermore, the protein expression levels of CD2AP (*P *= 0.0047) and nephrin (*P *= 0.0445) increased. In contrast, after 48 h of low‐oxygen culture and treatment with the HIF1α inhibitor, the protein expression levels of HIF1α (*P *= 0.0436) and KLF4 (*P *= 0.0192) in podocytes were reduced, and the protein expression levels of CD2AP (*P *= 0.0022) and nephrin (*P *= 0.0162) were also reduced compared with those in the hypoxic group (Figure 4).

### 3.5 Silencing of the KLF4 gene weakened the effect of HIF1α on podocytes under hypoxic conditions

**FIGURE 5:**
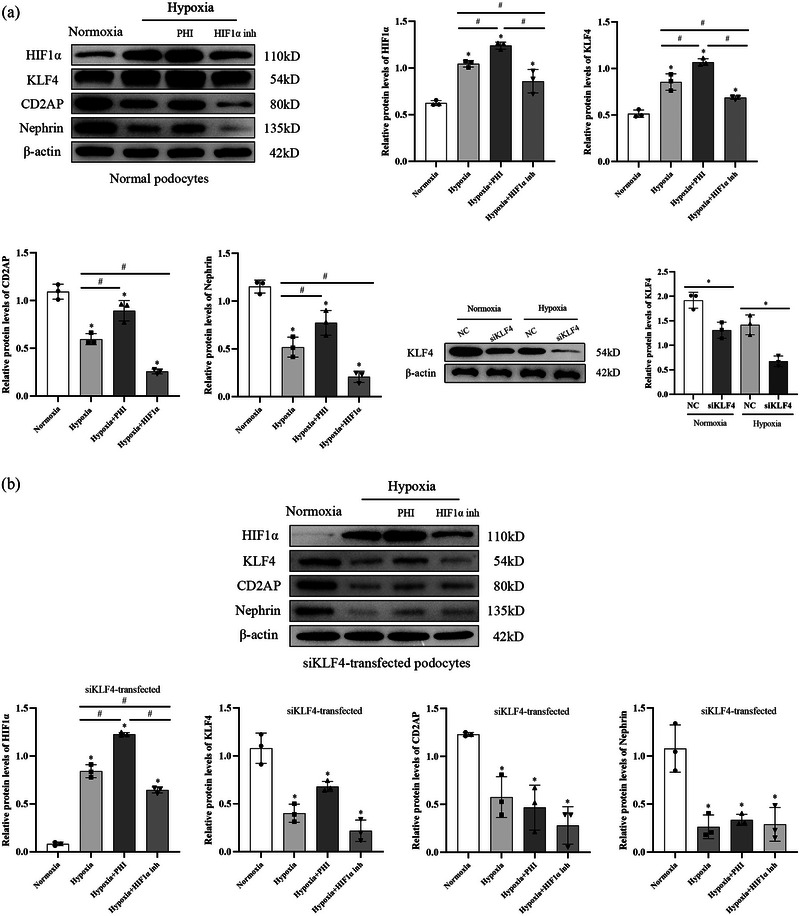
(a) Relative expression of KLF4 protein in podocytes with siKLF4 transfection. NC, non‐specific control group; siKLF4, siKLF4‐transfected group. The asterisk (*) indicates comparison with different groups. *n* = 3. Data are expressed as means ± SD. (b) Relative expression of HIF1α, KLF4, CD2AP and nephrin proteins in siKLF4‐transfected podocytes of each group: Normoxia group; Hypoxia group; Hypoxia + PHI group; Hypoxia + HIF1α inhibitor group. The asterisk (*) indicates comparison with normoxia group. The # symbol indicates comparison with different groups. *n* = 3. Data are expressed as means ± SD.

KLF4 protein expression in the siKLF4‐transfected podocytes under normoxic (*P *= 0.0074) and hypoxic (*P *= 0.0022) conditions was significantly lower than that in the non‐transfected group (Figure 5a), indicating that siKLF4 transfection effectively inhibited KLF4 protein expression. Compared to the normoxic podocytes, the siKLF4‐transfected podocytes subjected to 48 h of hypoxic culture showed significantly greater HIF1α (*P *< 0.0001) protein expression and lower CD2AP (*P *= 0.0012) and nephrin (*P *= 0.0014) protein expression. With the addition of PHI, the protein expression level of HIF1α in the siKLF4‐transfected podocytes cultured for 48 h under hypoxic conditions increased compared to that in the hypoxic group (*P *< 0.0001), while the protein expression levels of CD2AP (*P *= 0.8862) and nephrin (*P *= 0.9491) did not significantly differ from those in the hypoxic group. In contrast, with the addition of the HIF1α inhibitor, the siKLF4‐transfected podocytes cultured under hypoxic conditions for 48 h showed a decrease in HIF1α protein expression compared to that of the hypoxic group (*P *= 0.0013), while the expression levels of the CD2AP (*P *= 0.2837) and nephrin (*P *= 0.9975) proteins did not significantly differ from those in the hypoxic group (Figure 5b).

## 4 DISCUSSION

A hypobaric and hypoxic environment after rapid ascent to high altitudes can have certain effects on the body, including an impact on cardiac and pulmonary function, as well as the onset of proteinuria in some individuals (Wada et al., 2006; Talks et al., 2018). In the present study, rats reared at a simulated high altitude of 5000 m were found to have proteinuria with a molecular mass of approximately 70 kDa, accompanied by varying degrees of damage to the podocytes. There are trace amounts of protein in normal human urine, with a daily excretion of less than 150 mg. These urinary protein components are mainly small‐molecular proteins, such as β2‐microglobulin, urinary lysozyme and α1‐microglobulin. When the proximal renal tubules are damaged, the reabsorption of small‐molecular proteins decreases, and the level of urinary small‐molecular proteins increases. Podocytes play a key role in maintaining normal glomerular filtration, and podocyte damage contributes to proteinuria and is considered critical for the initiation and progression of chronic kidney disease (CKD). When podocyte damage occurs, medium molecular mass proteins, such as albumin, which has a molecular mass of approximately 70 kDa, appear in the urine.

In this study, the administration of PHI significantly decreased the level of medium molecular mass urine protein. This finding is consistent with our previous research (Zeng et al., 2022), which suggested that PHI may have a potential role in reducing proteinuria by upregulating local HIF1α expression in the kidney to alleviate podocyte injury. However, how does HIF1α achieve its protective effect on podocytes?

KLF4 belongs to the Krüppel‐like transcription factor family and is expressed in both the glomeruli and tubules of the kidney. This molecule plays an important role in the development and progression of many kidney diseases and is closely related to the occurrence of proteinuria (Ghaleb & Yang, 2017). Overexpression of KLF4 in cultured human podocytes can decrease the methylation of the promoters of podocyte epithelial markers such as nephrin and podocin, leading to changes in the podocyte phenotype (Hayashi et al., 2014). KLF4 may induce epigenetic changes that reverse podocyte injury by regulating the podocyte phenotype. Further animal experiments have shown that knockout of the *KLF4* gene in mice exacerbates albuminuria induced by adriamycin, while induction of *KLF4* gene expression in the kidney glomeruli either by tail vein injection or by podocyte‐specific transgenic repair can induce the recovery of podocyte epithelial markers such as nephrin and reduce proteinuria (Hayashi et al., 2014, 2015). These studies demonstrate that KLF4 is closely related to the repair of podocyte injury and the occurrence of proteinuria. To verify whether the protective effect of HIF1α on podocytes under hypobaric and hypoxic conditions is related to KLF4, we first observed the correlation between HIF1α expression and KLF4 expression in rats. This study used a hypobaric oxygen chamber to simulate an environment at an altitude of 5000 m. Under hypobaric and hypoxic conditions, the protein expression of HIF1α in rats initially increased and then decreased with prolonged hypoxia, while the change in the protein expression of KLF4 in the kidney was consistent with that of HIF1α. After the administration of PHI, the protein expression of HIF1α was upregulated, and the protein expression of KLF4 tended to increase. We found that after the administration of PHI, the urinary protein levels of the rats tended to decrease, with the most significant change observed on the 14th day of feeding. Moreover, the protein expression levels of CD2AP and nephrin were greater in the intervention group than in the experimental group, and the degree of podocyte injury detected by electron microscopy was also lower in the intervention group. CD2AP is an important protein on the pore membrane of podocytes. Mice with congenital deficiency of this protein exhibit a large amount of proteinuria shortly after birth, accompanied by fusion and disappearance of foot processes. The low expression of CD2AP may be involved in the pathogenesis of glomerular diseases characterized by podocyte lesions. CD2APs can interact with nephrin and transmit extracellular signals to cells. Abnormal expression of these molecules leads to the loss of normal podocyte morphology and the fusion and disappearance of foot processes, ultimately leading to the occurrence of proteinuria.

In vitro experiments involving hypoxic cell culture were further conducted. Rat podocytes were cultured in a hypoxic atmosphere with 1% O_2_, and cell growth was slow. After 48 h of hypoxic culturing, cell death was observed. When the culturing time exceeded 72 h, there was a significant increase in cell death. Therefore, subsequent in vitro experiments were conducted under 48 h of hypoxic (1% O_2_) culturing.

Compared with the podocytes cultured under normoxic conditions, the podocytes cultured under hypoxic conditions exhibited increased HIF1α and KLF4 protein expression and decreased CD2AP and nephrin protein expression. The addition of PHI to hypoxic‐cultured podocytes resulted in a more significant increase in the protein expression of HIF1α and KLF4, as well as an increase in the protein expression of CD2AP and nephrin. Conversely, in the hypoxic‐cultured podocytes treated with a HIF1α inhibitor, the protein expression of HIF1α and KLF4 decreased, and the protein expression of CD2AP and nephrin decreased. The HIF1α inhibitor (kc7f2) used in this study is a selective HIF1α protein translation inhibitor that inhibits HIF1α protein synthesis without affecting its mRNA transcription. To investigate whether the protective effect of HIF1α on podocyte damage is mediated by KLF4, we successfully inhibited the expression of KLF4 via siRNA. Regardless of whether the protein expression of HIF1α was upregulated or downregulated, podocyte lesions caused by hypoxia persisted, and the protective effect of HIF1α on podocytes was significantly weakened in the absence of KLF4. This finding suggested that the regulation of KLF4 expression may be one possible mechanism through which HIF1α participates in repairing podocyte lesions. Although evidence suggests that high levels of HIF1α during chronic hypoxia may lead to glomerular disease and proteinuria (Varga et al., 2018), this finding may be attributed to podocytes being more susceptible to damage during chronic hypoxia, including epithelial‐to‐mesenchymal transition, slit diaphragm dysfunction and disturbances in the cell cytoskeleton caused by HIF accumulation (Dong et al., 2021). This study indicated that under hypobaric and hypoxic conditions, HIF1α may induce KLF4 expression in the short term by intervening in podocyte epigenetics, thus alleviating podocyte injury and reducing proteinuria in rats.

There are several limitations to this study. First, in the animal experiments, only three time points—7, 14 and 28 days—were selected, and renal injury in rats in a low‐pressure and hypoxic environment was not explored for a short period of time—from a few hours to 1 day. Further research was not conducted on the physiological and pathological changes in rats after 28 days of hypoxia or even longer due to limitations in the experimental conditions. Second, with prolonged exposure to low pressure and hypoxia, the levels of medium molecular mass proteins in the urine of the rats in the experimental group first increased and then decreased, and the increase on day 14 was the most significant. Therefore, kidney tissue specimens were collected for western blot analysis and histopathological examination only on the 14th day. It was unclear whether there was a change in CD2AP and nephrin protein expression levels on the seventh day. In future research, kidney tissue specimens should be collected on day 7, day 14, and day 28. Thus, we could obtain a more complete understanding of the effect of PHI on rats under low‐pressure hypoxic conditions. Third, only PHI and HIF1α inhibitors were used to regulate HIF1α expression in this study. If RNA interference technology is used to silence HIF1α expression in vitro or gene knockout technology is used in animal experiments, the results could be more convincing. Fourth, changes in prolyl hydroxylase levels in rats under low‐pressure and hypoxic conditions were not observed in this study. It is unclear whether the treatments used during the study (HIF1 inhibition, KLF4 siRNA transfection) affect prolyl hydroxylase levels. This topic is worthy of further investigation. Finally, in the animal experiments, we chose all male rats. However, the excessive use of male animals may lead to bias in the experimental results due to the absence of sex differences.

## AUTHOR CONTRIBUTIONS

Cheng Yue and Zeng Yan conceived and designed the present study. Zeng Xiaoshan performed most of the animal intervention and subsequent experiments. Data interpretation was done by Mo Liwen and Gan Zhilin. Initial manuscript draft was written by Zeng Xiaoshan, with subsequent by revision by Cheng Yue and Cheng Huan. All authors have read and approved the final version of this manuscript and agree to be accountable for all aspects of the work in ensuring that questions related to the accuracy or integrity of any part of the work are appropriately investigated and resolved. All persons designated as authors qualify for authorship, and all those who qualify for authorship are listed.

## CONFLICT OF INTEREST

The authors declare no conflicts of interests or financial interests.

## Data Availability

Upon reasonable request, the analysed datasets are available from the corresponding author.
